# Ethanol Production by Selected Intestinal Microorganisms and Lactic Acid Bacteria Growing under Different Nutritional Conditions

**DOI:** 10.3389/fmicb.2016.00047

**Published:** 2016-01-29

**Authors:** Fouad M. F. Elshaghabee, Wilhelm Bockelmann, Diana Meske, Michael de Vrese, Hans-Georg Walte, Juergen Schrezenmeir, Knut J. Heller

**Affiliations:** ^1^Department of Microbiology and Biotechnology, Max Rubner-Institut (Federal Research Institute of Nutrition and Food)Kiel, Germany; ^2^Department of Dairy Science, Faculty of Agriculture, Cairo UniversityGiza, Egypt; ^3^Department of Safety and Quality of Milk and Fish, Max Rubner-Institut (Federal Research Institute of Nutrition and Food)Kiel, Germany; ^4^Medical Clinic, Johannes Gutenberg UniversityMainz, Germany

**Keywords:** *Weissella confusa*, fructose, arabinose, lactulose, inulin, ethanol, fecal slurries, non-alcoholic fatty liver disease

## Abstract

To gain some specific insight into the roles microorganisms might play in non-alcoholic fatty liver disease (NAFLD), some intestinal and lactic acid bacteria and one yeast (*Anaerostipes caccae, Bacteroides thetaiotaomicron, Bifidobacterium longum, Enterococcus fecalis, Escherichia coli, Lactobacillus acidophilus, Lactobacillus fermentum, Lactobacillus plantarum, Weissella confusa, Saccharomyces cerevisiae*) were characterized by high performance liquid chromatography for production of ethanol when grown on different carbohydrates: hexoses (glucose and fructose), pentoses (arabinose and ribose), disaccharides (lactose and lactulose), and inulin. Highest amounts of ethanol were produced by *S. cerevisiae, L. fermentum*, and *W. confusa* on glucose and by *S. cerevisiae* and *W. confusa* on fructose. Due to mannitol-dehydrogenase expressed in *L. fermentum*, ethanol production on fructose was significantly (*P* < 0.05) reduced. Pyruvate and citrate, two potential electron acceptors for regeneration of NAD^+^/NADP^+^, drastically reduced ethanol production with acetate produced instead in *L. fermentum* grown on glucose and *W. confusa* grown on glucose and fructose, respectively. In fecal slurries prepared from feces of four overweight volunteers, ethanol was found to be produced upon addition of fructose. Addition of *A. caccae, L. acidophilus, L. fermentum*, as well as citrate and pyruvate, respectively, abolished ethanol production. However, addition of *W. confusa* resulted in significantly (*P* < 0.05) increased production of ethanol. These results indicate that microorganisms like *W. confusa*, a hetero-fermentative, mannitol-dehydrogenase negative lactic acid bacterium, may promote NAFLD through ethanol produced from sugar fermentation, while other intestinal bacteria and homo- and hetero-fermentative but mannitol-dehydrogenase positive lactic acid bacteria may not promote NAFLD. Also, our studies indicate that dietary factors interfering with gastrointestinal microbiota and microbial metabolism may be important in preventing or promoting NAFLD.

## Introduction

Non-alcoholic fatty liver disease (NAFLD) is defined as excessive accumulation of triglycerides in the liver of humans, who do not consume large amounts of alcohol (<20 g ethanol per day) (McCullough, 2006; WGO, 2012). It is prevalent in obese and diabetic subjects (Wanless and Lentz, 1990; Bellentani et al., 1994; Clark et al., 2002; Chitturi et al., 2004). Gut microbiota has been described to promote NAFLD and non-alcoholic steatohepatitis (NASH) by different mechanisms including small intestinal bacterial overgrowth (SIBO), and impaired intestinal barrier integrity, which result in increased endotoxin (LPS) translocation and progression of NAFLD to NASH (Solga and Diehl, 2003).

The human gut microbiota harbors between 10^13^ and 10^14^ microorganisms, which belong to over 1000 known species. Dominant bacterial phylotypes are *Firmicutes, Bacteroidetes*, and *Actinobacteria* (Egert et al., 2006; Gill et al., 2006). Changes in the gut microbiota were observed in obese and NAFLD patients in comparison with normal subjects. Increased total lactobacilli and decreased *Bacteroidetes* counts were associated with obesity, while increased counts were associated with leanness (Armougom et al., 2009). Some *Bifidobacterium* or *Lactobacillus* species were associated with normal weight (*Bifidobacterium animalis*) while *Lactobacillus reuteri* was associated with obesity (Million et al., 2012). A patient with a history of nonalcoholic steatohepatitis (NASH), liver transplantation, hepatic artery stent and biliary stent showed *Weissella confusa* bacteremia (Harlan et al., 2011). Ethanol is one of the dominating metabolites of hetero-lactic intestinal microbes. On the basis of histologic features for obesity-related and alcohol-induced liver damage, Cope et al. (2000) proposed that ethanol produced by the gut microbiota could be responsible for NAFLD. This idea was further discussed by Baker et al. (2010). According to their hypothesis, increased ethanol production by a dysbalanced gut microbiota of obese subjects on the one hand and elevated NADH levels in the liver on the other hand induce expression of cytochrome P450 2E1, which through generation of reactive oxygen species (ROS) causes liver inflammation.

The gut microbiota plays an important role in energy harvest of the host. Energy harvest and fat deposition were lower and fecal excretion of calories was higher in germ free rats than in conventional rats (Wostmann et al., 1983). Short chain fatty acids in caecum and small intestine of conventional mice were 75–5000 times higher than those in caecum and intestine of germ-free mice (Høverstad and Midtvedt, 1986). Short chain fatty acids and butyrate in particular play important roles as energy substrates for peripheral tissues and the colonic epithelium, and *Bacteroides* ssp. and e.g., *Anaerostipes caccae* appear to be important gut bacteria involved in production of short chain fatty acids (Bergman, 1990). Prebiotic substances like lactulose and inulin were shown to promote growth of bifidobacteria in a rat model of NASH or in diet-induced obese mice, which correlated with reduced levels of lipopolysaccharides (LPS) (Fan et al., 2005; Cani et al., 2007). LPS is supposed to be involved in inducing liver inflammation (Bergheim et al., 2008; Baker et al., 2010). Recently, inulin has been shown to prevent development of hepatic steatosis in a rat model (Sugatani et al., 2012).

Increased consumption of carbohydrates like sucrose, glucose, and fructose has been correlated with traits of the metabolic syndrome diseases like obesity, steatosis, or insulin resistance (Gross et al., 2004; Solga et al., 2004; Libuda et al., 2008). Replacement of glucose for fructose caused accumulation of fat in the liver tissues (Bergheim et al., 2008). In contrast to glucose, fructose metabolism is insulin-independent (Lim et al., 2010). Since it does not yield a level of satiety comparable to that of glucose, it is more involved in induction of obesity (Machado and Cortez-Pinto, 2012). Furthermore, intestinal uptake of fructose is different from that of glucose. GLUT5 has been identified as the major intestinal transport system for fructose (Douard and Ferraris, 2008). Compared to glucose, fructose uptake is limited, resulting in transit to the colon when overdosed. In 50% of the tested subjects hydrogen production by gastrointestinal microbiota increases when 30–40 g are ingested, indicating that fructose is not fully absorbed in the small intestine and enters the large intestine where it is metabolized by the microbiota (Jones et al., 2011).

The aim of our investigation was to evaluate ethanol production of some gastrointestinal bacteria and a yeast strain under different conditions, and to discuss the results obtained with respect to the alcohol hypothesis of NAFLD. For this, we investigated ethanol production of several defined, intestinal microorganisms growing on different carbohydrate sources. Those organisms with rather high or consistent ethanol production were tested for the effects of dietary electron acceptors (pyruvate and citrate) on ethanol production from glucose and fructose. Finally, some of the microorganisms applied were tested for their effects on fructose fermentation by fecal slurries prepared from stool samples of four healthy overweight volunteers.

## Materials and methods

### Microbes and growth conditions

*A. caccae* DSM 14662 (Schwiertz et al., 2002) and *Bacteroides thetaiotaomicron* DSM 94168 were obtained from DSMZ GmbH, Braunschweig, Germany. *Bifidobacterium* (*Bi*.) *longum* NRRL-B- 41409, *Lactobacillus* (*L*.) *acidophilus* NRRL-B-4495 and *Weissella* (*W*.) *confusa* NRRL-B- 14171 were obtained from Northern Regional Research Laboratory (NRRL), Peoria, USA. *W. confusa* was provided by NRRL as *L. reuteri* NRRL-B-14171; however, 16S rDNA sequencing clearly identified it as *W. confusa*. *L. plantarum* 92380, *Enterococcus* (*Ec*.) *fecalis* 90561, *Escherichia* (*E*.) *coli* 98082, *L. fermentum* 92294, and *Saccharomyces* (*S*.) *cerevisiae* 56101 were from the culture collection of Department of Microbiology and Biotechnology at Max Rubner-Institute (Kiel, Germany). All tested microbial strains were isolated from human intestine except *Ec. fecalis, L. fermentum*, and *S. cerevisiae*, which were isolated from raw milk, Kenyan fermented product Kimere, and Harzer cheese, respectively.

*A. caccae* was sub-cultured in PYG medium without resazurin (DSMZ 104: http://www.dsmz.de/microorganisms/medium/pdf/DSMZ_Medium104.pdf) and incubated at 37°C for 48 h. *B. thetaiotaomicron* and *Bi. longum* were sub-cultured in Wilkins Chalgren broth (Wilkins and Chalgren, 1976) at 37°C for 48 h. *L. acidophilus, L. fermentum, L. plantarum*, and *W. confusa* were propagated in deMan, Rogosa, Sharp (MRS) medium (De Man et al., 1960). *Ec. fecalis* 90561 was sub-cultured in M17 medium (Terzaghi and Sandine, 1975) and *E. coli* was propagated in Nutrient broth (Eaton et al., 1995). All lactobacilli, *Ec. fecalis* and *E. coli* were incubated at 37°C for 24 h. *S. cerevisiae* was propagated in Malt extract broth (Galloway and Burgess, 1952) and incubated aerobically at 25°C for 48 h. Anaerobic incubations were carried out in an anaerobic chamber at 37°C.

Wilkins Chalgren broth and M17 medium were purchased from Oxoid, (London, UK)., Malt extract broth, nutrient broth, Phenol, sulfuric acid, arabinose, and fructose were purchased from Merck Co. (Darmstadt, Germany).

Glucose, and lactose (4-*O*-(β-D-Galactopyranosyl)-D-glucopyranose) were purchased from Carl Roth GmbH (Karlsruhe, Germany). Lactulose (4-*O*-β-D-Galactopyranosyl-D-fructofuranose) was purchased from Molekula Co. (Dorset, UK), D-(-)-ribose and inulin (polyfructose with one terminal glucose moiety, *n* = ca. 35) from Fluka Co. (Virginia, USA).

In experiments with fecal slurries, neomycin was added at concentrations of 1 mg/ml (Cope et al., 2000).

“Roti-Store Cryoröhrchen” tubes for culture storage at −80°C were purchased from Carl Roth GmbH Co. (Karlsruhe, Germany).

The anaerobic chamber used was from Don Whitley Scientific Limited (West Yorkshire, UK).

Metacarb 87H HPLC column was purchased from VWR international Co. (Darmstadt, Germany). High liquid performance chromatograph (HPLC) machine was purchased from Merck-Hitachi (Darmstadt, Germany).

### Culture preservation

Activated cultures of single microbial strains were streaked on growth media containing 1.5% agar. After incubation for 48 h at 37°C for bacterial strains or 72 h at 25°C for yeast strain, the colonies were swapped after microscopic examination, transferred to Roti-Store Cryo tubes and stored at −80°C.

### Fermentation in media for colonic bacteria (MCB)

Fermentations were performed in media for colonic bacteria (MCB) at 37°C in an anaerobic chamber. The medium composition (gL^−1^) was bacteriological peptone, 6.5; soy peptone, 5.0; tryptone, 2.5; yeast extract, 3.0; KCl, 2.0; NaHCO_3_, 0.2; NaCl, 4.5; MgSO_4_ · 7H_2_O, 0.5; CaCl_2_ · 2H_2_O, 0.45; MnSO4 · H_2_O, 0.2; FeSO4 · 7H_2_O, 0.005; ZnSO4 · 7H_2_O, 0.005; cysteine-HCl,0.4; hemin, 0.005; menadion, 0.005. One liter medium contained 0.5 ml H_3_PO_4_ and 2 ml Tween 80. The pH of the medium was adjusted to 5.8 before sterilization (121°C, 20 min). Glucose, fructose, lactose, lactulose, arabinose, ribose or inulin were used as sole energy sources and separately and aseptically added to the fermentation medium, at concentrations of 15 g L^−1^. All sugars were sterilized in an autoclave (210 kPa, 121°C, 20 min) except ribose and arabinose, which were sterilized by filtration through 0.2 μm membrane filters (Van der Meulen et al., 2006a). Inoculation ratio was 5% for each tested strain. Growth was monitored by measuring optical density at 620 nm (OD_620_) after incubation at 37°C for 48 h for *A. caccae* DSM 14662^T^ and *Ba. thetaiotaomicron* DSM 94168 and for 24 h for all other strains. Determination of inulin in microbiological media was done by modified phenol sulfuric acid method using inulin as standard (Dubois et al., 1956; Masuko et al., 2005).

Dietary electron acceptors citrate and pyruvate were added in concentrations of 7.5 and 15 g l^−1^, respectively.

### Enumeration of microorganisms after fermentation

At the end of fermentation, 1 ml of each sample was serially diluted in phosphate-buffered saline (0.14 M NaCl, 2.7 mM KCl, 8.1 mM Na_2_HPO_4_, 1.8 mM KH_2_PO_4_; pH 7.4) in 1:10 steps. One hundred microliters of appropriate dilutions were plated on suitable media: MRS medium supplemented with 1.5% agar was used for enumeration of *W. confusa* and *L. fermentum*. Plates were incubated anaerobically at 37°C for 3 days. *S. cerevisiae* was enumerated by plating on malt extract agar plates supplemented with 20 ml of 10% lactic acid solution per Liter. Plates were incubated at 25°C for 5 days.

### Metabolites analyses

**Figure 1:**
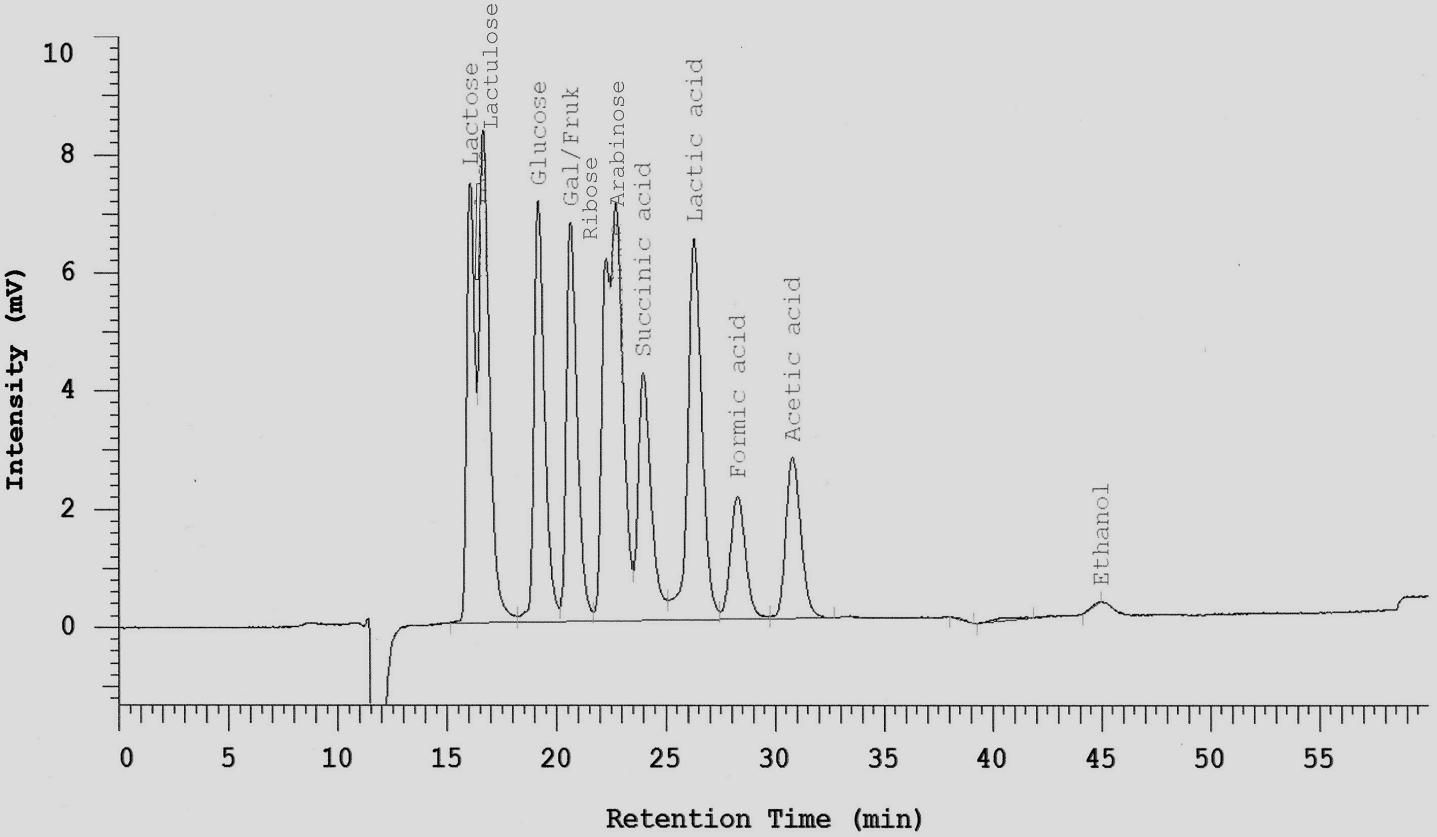
**Separation of selected sugars, acids, and ethanol on a Metacarb 87H column (7.5 × 300 mm) with 0.0085 N H_**2**_SO_**4**_ at a flow rate of 0.3 ml/min and 65°C**.

One mL of each tested cultures was mixed with 10 μL Carrez I and 10 μL Carrez II solution and centrifuged at 14,000 × g for 10 min at 4°C. The clarified layer was separated and filtered through 0.2 μm membrane filter. All metabolite samples were frozen at -20°C until analysis. Sugars (hexose, disaccharides, and pentose), lactate, succinate, short chain fatty acids (acetate, butyrate and propionate), acetaldehyde, and ethanol were determined by HPLC after dilution of samples with sulfuric acid 0.0085 N (1:25) on a Metacarb 87H column 7.5 × 300 mm. The mobile phase was sulfuric acid 0.0085 N with a flow rate of 0.3 mL/min. The Metacarb HPLC column was coupled with refractive index (RI) detector was heated at temperature 65°C. Appropriate standards were used for the identification of metabolites (an example is shown in Figure 1). For better separation of glycerol in fermentations with *S. cerevisiae*, 0.1 N H_2_SO_4_ was used as mobile phase with a flow rate of 0.3 ml/min. Retention times for the different sugars, acids and alcohols are listed in Table 1.

**Table 1:** **Retention times of different sugars, acids, and alcohols separated on Metacarb 87H columns**.

**Sugars/Acids/Alcohols**	**Retention time [min]**
**Mobile phase: 0.0085 N H**_2_**SO**_4_**, 0.3 ml/min**	
Lactose	16.31
Na-Citrate	16.32
Lactulose	16.79
Na-Pyruvate	18.32
Glucose	19.33
Fructose/Galactose	20.77
Mannitol	21.45
L-Arabinose	22.37
Ribose	22.87
Succinic acid	23.99
Erythritol	24.35
Succinic acid	25.07
Lactic acid	26.45
Formic acid	28.43
Acetic acid	30.99
Propionic acid	35.96
Acetaldehyde	39.00
Butyric acid	43.85
Ethanol	45.48
**Mobile phase: 0.1 N H**_2_**SO**_4_**, 0.6 ml/min**	
Glucose	11.00
Lactic acid	15.48
Glycerol	16.30
Acetic acid	17.95

### Preparation of fecal slurries

Fecal samples from four healthy, male, obese volunteers were collected in sterilized plastic bags, which were sealed immediately after removal of as much air as possible. Volunteers had a body mass index (BMI) between 28 and 33. They had no history of antibiotics treatment for the last 6 months and no history of alcohol consumption. The bags were transported to the laboratory at ambient temperature and further processed. Twenty grams of feces samples were suspended in 100 mL anaerobic phosphate-buffered saline (0.14 M NaCl, 2.7 mM KCl, 8.1 mM Na_2_HPO_4_, 1.8 mM KH_2_PO_4_, 0.05% L-cysteine HCl, and 0.2% Tween 80; pH 7.4). Two milliliters of homogenized slurry were inoculated in 10 mL of MCB medium supplemented with fructose, fructose with citrate, fructose with pyruvate; fructose with neomycin, respectively, followed by incubation at 37°C under anaerobic conditions in anaerobic chamber for 24 h. The concentrations of supplements were: fructose 15 mg/ml, pyruvate 15 mg/ml; citrate 7.5 mg/ml; neomycin 1 mg/ml. Also, 1 mL of homogenized slurry as well as 1 ml of overnight single strain cultures of *A. caccae, L. acidophilus, L. fermentum*, or *W. confusa*, respectively were inoculated in 10 mL of MCB supplemented with fructose under the same conditions.

### Enumeration of different bacterial groups in fresh feces and fecal slurry samples

One g of fresh feces sample was suspended in 99 mL Ringer solution (6.5 g NaCl, 0.42 g KCl, 0.25 g CaCl_2_, and 0.2 g of NaHCO_3_) supplemented with 0.05% L-cystein HCL, followed by serial 1:10 dilution in Ringer solution. One hundred microliters of appropriate dilutions were spread on different selective media and incubated anaerobically as follows: total anaerobic bacteria [brain heart infusion agar (Merck, Darmstadt, Germany), 37°C/48 h]; total lactobacilli [LAMVAB agar Hartemink et al. (1997), 37°C/72 h], total *Enterobacteriaceae* [crystal violet agar (Merck, Darmstadt, Germany), 37°C/24 h; total clostridia (reinforced clostridial agar, Becton and Dickinson, Heidelberg, Germany) 37°C/48 h].

### Alcohol dehydrogenase activity

Alcohol dehydrogenase activities (ADH) in fecal slurries were determined at the end of fermentation period (after 24 h) by “Alcohol Dehydrogenase Detection Kit ab102533” of Abcam® (Cambridge, UK), according to the manual provided by the supplier. The test relies on the interconversion of NADH to NAD^+^ in the presence of alcohol. The amount of NAD+ formed can be measured colorimetrically at 450 nm and—when compared to a standard curve—can be used to calculate ADH activity. ADH activities as low as 0.01 mU can be detected.

### Statistical analyses

Data are expressed as means of three replicates ± standard deviation (SD). Statistical analysis was carried out with SAS software (version 9.2). Tukey's *post-hoc* test (*P* < 0.05) was used for evaluating the influence of different bacterial species, dietary electron acceptors, and neomycin on ADH activities in different fecal slurries samples.

## Results and discussion

Ten microorganisms were screened for ethanol production in this study. *A. caccae* DSM 14662, *Ba. thetaiotaomicron* DSM 94168, *B. longum* NRRL-B- 41409, *E. coli* 98082, *L. acidophilus* NRRL B-4495. *L. plantarum* 92380, and *W. confusa* NRRL-B-14171 were intestinal isolates, while *Ec. fecalis* 90561, *L. fermentum* 92294, and *S. cerevisiae* 56101 were isolates from foods. The strains represented the bacterial phyla *Actinobacteria* (*Bifidobacterium*), *Bacteroidetes* (*Bacteroides*), *Firmicutes* (*Anaerostipes, Enterococcus, Lactobacillus, Weissella*), and *Proteobacteria* (*Escherichia*) as well as yeasts (*Saccharomyces*).

### Screening for ethanol production of microbial strains growing of different carbohydrates

Growth of the 10 microbial strains on different carbohydrate sources was tested by measuring optical densities (OD_620_), pH-values and residual carbohydrate remaining at the end of fermentation after 24 of incubation (48 h for *A. caccae* DSM 14662 and *Ba. thetaiotaomicron* DSM 94168) (Table 2). In addition, major metabolites produced are shown. All 9 tested bacterial strains were able to ferment glucose, fructose, lactose, and lactulose. Arabinose was not fermented by *L. acidophilus* and *W. confusa*, ribose was not fermented by *B. thetaiotaomicron* DSM 94168, and inulin not by *A. caccae DSM 14662* and *E. coli*. *S. cerevisiae* was able to ferment glucose and fructose only.

**Table 2:** **Growth and metabolic features at 37°C in MCB medium supplemented with different carbohydrates determined by measuring ΔOD620, pH, carbohydrate consumed, and major metabolite**.

**Microbial species**	**ΔOD620** **Final pH** **Carbohydrate consumed (mM)** **Major metabolite**						
	**Glc**	**Fru**	**Ara**	**Rib**	**Lac**	**Lacl**	**Inu**
*A*. *caccae* DSM 14662	1.6±0.3^1^	1.4±0.3	0.8±0.3	1.5±0.3	0.7±0.3	0.5±0.3	0
	4.4±0.1	4.4±0.1	5.2±0.1	4.5±0.1	5.2±0.1	5.0±0.1	5.8±0.1
	43.3±2.1	42.3±2.5	9.0±1.0	49.3±1.0	26.3±2.3	25.7±2.0	0
	But	But	But	But	But	But	–
*B*. *thetaiotaomicron* DSM 94168	1.8±0.2	1.8±0.2	0.9±0.3	0	1.1±0.1	1.2±0.1	1.5±0.3
	4.2±0.1	4.2±0.1	4.8±0.1	5.8±0.1	4.6±0.1	4.6±0.1	3.9±0.1
	52.9±2.0	49.8±2.0	20.4±2.3	1.9±0.5	22.4±1.3	21.5±1.0	1.6±0.5
	Succ	Succ	Succ	–	Ac∕Succ	Ac	Succ
*Bi. longum* NRRL-B- 41409	2.2±0.2	2.0±0.3	0.8±0.4	0.8±0.3	0.8±0.3	0.9±0.4	0.5±0.3
	3.8±0.1	4.0±0.1	4.0±0.1	4.4±0.1	4.2±0.1	4.1±0.2	4.8±0.2
	52.4±3.0	42.8±2.0	27.7±2.0	27.4±0.6	19.4±1.0	28.5±1.0	0.9±0.2
	Ac	Ac	Lact	Lact	Ac∕Lact	Ac∕Lact	Ac
*E*. *coli* 98082	1.3±0.3	1.1±0.3	0.9±0.3	0.8±0.3	0.9±0.2	0.9±0.3	0
	4.0±0.1	4.0±0.1	4.8±0.1	4.8±0.1	4.3±0.1	4.4±0.1	5.3±0.1
	44.3±2.0	44.8±0.8	11.0±2.0	11.0±0.7	17.5±1.0	17.5±1.0	0
	Lact	Lact	Ac∕Lact	Ac∕Lact	Lact	Lact	–
*Ec. faecalis* 90561	1.5±0.1	1.0±0.2	0.6±0.2	1.1±0.3	1.1±0.1	0.9±0.3	0.9±0.3
	4.0±0.1	4.0±0.1	5.0±0.1	4.1±0.1	4.4±0.1	4.6±0.1	4.7±0.1
	20.9±3.0	20.3±1.0	10.0±1.5	50.6±0.6	11.4±1.3	12.5±1.0	0.6±0.2
	Lact	Lact	Lact	Lact	Lact	Lact	Lact
*L*. *acidophilus* NRRL-B-4495	1.1±0.1	1.0±0.2	0	0.3±0.2	1.3±0.2	1.0±0.2	0.8±0.2
	3.5±0.1	3.6±0.1	5.6±0.1	4.3±0.1	3.6±0.1	3.8±0.1	4.3±0.1
	42.3±2.0	40.3±2.6	1.9±0.7	32.9±0.8	26.4±3.1	25.4±2.0	0.7±0.4
	Lact	Lact	–	Ac∕Lact	Lact	Lact	Lact
*L. plantarum* 92380	5.9±0.3	5.3±0.3	1.6±0.4	1.5±0.3	5.2±0.3	5.6±0.2	0.8±0.3
	3.2±0.1	3.2±0.1	3.8±0.1	4.4±0.1	3.4±0.1	3.2±0.1	4.3±0.1
	55.3±2.0	55.0±1.5	25.7±3.0	24.9±2.0	14.5±1.8	13.3±2.0	1.2±0.2
	Lact	Lact	Ac∕Lact	Ac∕Lact	Lact	Lact	Lact
*L*. *fermentum* 92294	6.3±0.3	4.0±0.2	1.8±0.3	1.7±0.1	2.5±0.1	2.8±0.1	0.3±0.2
	3.4±0.1	3.9±0.1	3.2±0.1	4.0±0.1	4.5±0.1	3.5±0.1	5.3±0.1
	83.3±0.0	83.3±0.0	34.4±3.0	32.9±2.3	13.3±2.0	43.1±0.5	0.3±0.2
	Eth∕Lact	Lact∕Man	Ac∕Lact	Ac∕Lact	Ac∕Lact	Ac∕Lact	Lact
*W. confusa* NRRL-B- 14171	1.9±0.3	1.6±0.2	0	1.3±0.2	0.6±0.3	0.5±0.3	0.5±0.3
	3.4±0.1	3.6±0.1	n.d.	3.8±0.1	5.0±0.1	4.9±0.1	4.8±0.1
	49.3±1.7	49.7±1.7	1.9±1.0	65.4±2.4	11.6±2.0	11.3±2.3	0.3±0.2
	Eth∕Lact	Eth∕Lact	–	Ac∕Lact	Ac∕Lact	Ac∕Lact	Ac∕Lact
*S. cervisiae* 56101	1.7±0.4	1.0±0.3	0	0	0	0	0
	5.3±0.1	5.4±0.1	5.8±0.1	5.8±0.1	5.8±0.1	5.8±0.1	5.8±0.1
	78.8±3.2	70.9±3.0	0	0	0	0	0
	Eth	Eth	–	–	–	–	–

*Initial pH of MCB medium was 5.8 and initial concentrations (mM) of carbohydrates were 83.3 for hexoses, 99.9 for pentoses, 43.8 for disaccharides, and 3.0 for inulin. Glc, glucose; Fru, fructose; Lac, lactose; Lacl, lactulose; Rib, ribose; Ara, arabinose; Inu, inulin. Ac, acetate; But, butyrate; Eth, ethanol; Lact, lactate; Succ, succinate. n.d., no data; -, no product*.

1 *Data are presented as means ± standard deviation*.

**Figure 2:**
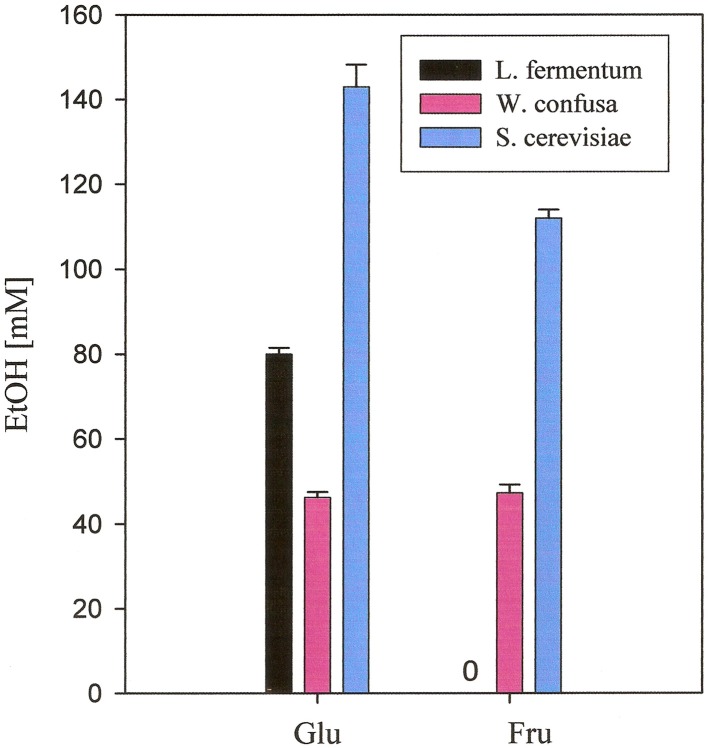
**Ethanol production from glucose and fructose, respectively, of microorganisms grown anaerobically in MCB-medium at 37°C**. Black, *L. fermentum* 92294; pink, *W. confusa* NRRL-B-14171; blue, *S. cerevisiae* 56101.

No ethanol at all was produced by *A. caccae, Ec. fecalis, L. acidophilus*, and *L. plantarum*. Small amounts were produced by *Ba. thetaiotaomicron* DSM 94168, *Bi. longum* NRRL- B- 41409, and *E. coli* from hexoses and from inulin by *Ba. thetaiotaomicron* DSM 94168. Significant production of ethanol by the remaining three organisms is documented in Figure 2. *L. fermentum* produced high amounts from glucose and no ethanol from the other carbohydrates. *W. confusa* produced rather high amounts from glucose and fructose. Highest ethanol production was observed for *S. cerevisiae* from glucose and fructose, the two carbohydrates on which this organism was able to grow on. Analyses of other metabolites (not shown) indicated and confirmed known metabolic activities of the microorganisms tested. Gases, however, were not analyzed.

*A. caccae*, one of the most abundant bacterial groups in the human colon (Falony et al., 2009), has been described as a butyrate-producing intestinal microorganism (Schwiertz et al., 2002; Duncan et al., 2004). To our knowledge, pentose metabolism has not yet been described for *A. caccae*. Our data indicate that both pentoses applied are metabolized, whereby ribose is more than arabinose. Butyrate appears to be the dominating product (Table 2).

*B. thetaiotaomicron*, a bacterium well known as an intestinal organism, has been described to produce mainly succinate, acetate, and propionate from anaerobic glucose fermentation (Kotarski and Salyers, 1981; Van der Meulen et al., 2006a). Degnan and MacFarlane (1995) showed that hexoses were more efficiently metabolized than pentoses. These reports are supported by the data presented in Table 2: we found succinate to be the dominating metabolite, with the exception of the two disaccharides lactose and lactulose where acetate/succinate and acetate, respectively, were dominating.

Bifidobacteria are among the most abundant intestinal microorganisms: they can account for up to ca. 3% of the human intestinal microbiota (Van der Meulen et al., 2006b). Their sugar metabolism has been found to mainly produce acetate and lactate, however, production of ethanol has also been described (Van der Meulen et al., 2006a,b; Amaretti et al., 2007; Margolles and Sánchez, 2012). Pentoses are readily fermented as well as inulin (Tamime, 2002). This is in agreement with our findings. Table 2 shows that acetate and lactate were produced from hexoses and pentoses, respectively, whereas lactate was the dominating metabolite from the disaccharides tested. For inulin, we found that mainly acetate was produced.

*E. coli* is a member of the family *Enterobacteriaceae*, which typically anaerobically degrade carbohydrates by mixed acid fermentation (Böck and Sawers, 1996). Ethanol is one of the common end products of this pathway. Pentose metabolism in *E. coli* has been described to occur via pentose phosphate pathway (Fraenkel, 1996). Our data (Table 2) incompletely reflect the heterogeneity of metabolites produced. With the exception of pentoses, where actetate and lactate were mainly produced, lactate appears constantly to be the only major metabolite produced.

*Ec. fecalis* and *L. acidophilus* are both members of the order *Lactobacillales*. They are known to metabolize hexoses via homo-lactic fermentation (Tamime, 2002; Pot and Tsakalidou, 2009), which explains that no ethanol is produced from hexoses, at least under conditions of sufficient substrate supply (Buckel, 1999). In our experiments, lactate was found to be the major metabolite with one exception: *L. acidophilus* mainly produced acetate together with lactate from ribose (Table 2).

*L. plantarum* is a facultative hetero-lactic organism (Pot and Tsakalidou, 2009). Hexoses are metabolized via homo-lactic fermentation basically yielding lactic acid only. Pentoses are metabolized via hetero-lactic fermentation yielding lactic acid, carbondioxide, and acetic acid (Buckel, 1999). Our results are in agreement with this (Table 2).

*L. fermentum* and *W. confusa* are both hetero-lactic organisms (Collins et al., 1993; Pot and Tsakalidou, 2009) known to produce lactic and acetic acid from pentoses and lactic acid, carbondioxide, and ethanol from hexoses (Buckel, 1999). However, *L. fermentum* belongs to the group of hetero-lactic lactobacilli expressing the enzyme mannitol dehydrogenase, which allows these organisms to use for NAD(P)^+^ regeneration fructose directly by reduction to mannitol (Maicas et al., 2002). Under these conditions, acetic acid is produced from hexoses instead of ethanol. Our data (Table 2) show that indeed ethanol together with lactate is the major metabolite of both organisms when fermenting glucose. Fructose fermentation yielded ethanol in high amount only for *W. confusa* NRRL-B-14171, whereas for *L. fermentum* 92294 lactate and mannitol were found to be produced in almost equimolar amounts. Fermentation of disaccharides resulted in equimolar amounts of acetate and lactate in both organisms. The same major metabolites were also produced from both pentoses by *L. fermentum* 92294 and by *W. confusa* NRRL-B-14171 from ribose. The latter organism did not utilize arabinose (Table 2).

Finally, *S. cerevisiae*, typically for yeasts, metabolizes hexoses via ethanol fermentation, yielding just ethanol and carbondioxide (Buckel, 1999). While this was confirmed in our experiments for ethanol (carbondioxide was not analyzed), the pentoses and disaccharides tested as well as inulin were not fermented at all (Table 2).

### Effects of dietary electron acceptors on ethanol production of *L. fermentum* 92294, *W. confusa* NRRL-B-14171, and *S. cerevisiae* 56101

**Figure 3:**
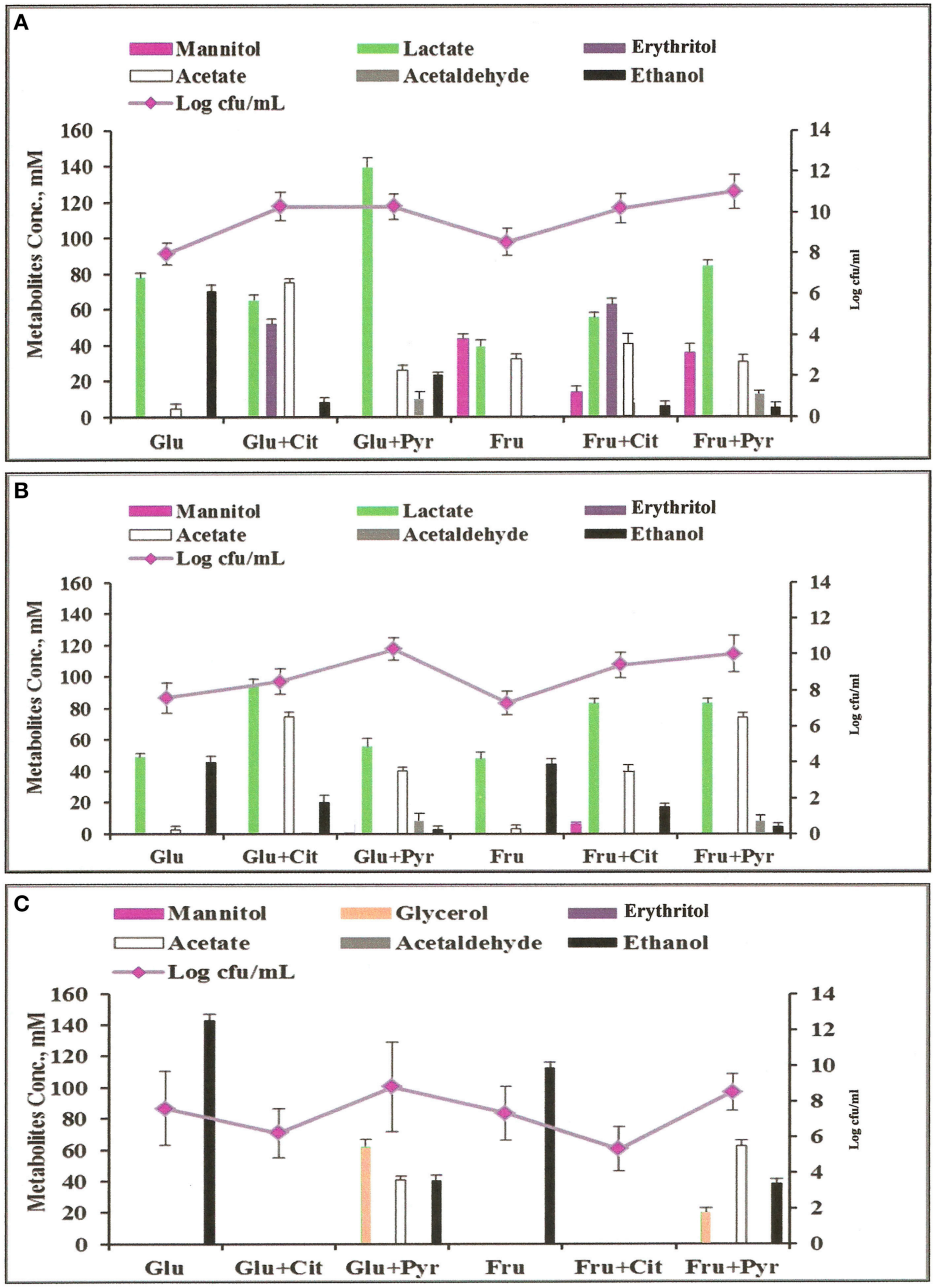
**Metabolites profiles and colony forming units (cfu) of microorganisms grown anaerobically in MCB medium at 37°C with different supplements: Glu, glucose; Fru, fructose; Cit, citrate; Pyr, pyruvate**. **(A)** *L. fermentum* 92294; **(B)** *W. confusa* NRRL-B-14171; **(C)** *S. cerevisiae* 56101.

The three significantly ethanol producing microorganisms *L. fermentum* 92294*, W. confusa* NRRL-B-14171, and *S. cerevisiae* 56101 were chosen to evaluate the effects of dietary electron acceptors citrate and pyruvate on ethanol production. Maicas et al. (2002) had shown that external electron acceptors like fructose or pyruvate were able to compensate for a defect in NAD(P)H regeneration through ethanol production caused by fermentation under aerobic conditions. Since we had already seen (Table 2) that *L. fermentum* 92294 but not *W. confusa* NRRL-B-14171 was able to apply fructose as electron acceptor (under formation of mannitol) comparison of these two organisms appeared meaningful. In addition, *S. cerevisiae* was tested, because it produced high amounts of ethanol. All three organisms were kept at 37°C in MCB medium with the following supplements: glucose, glucose plus citrate, glucose plus pyruvate, fructose, fructose plus citrate, fructose plus pyruvate. After 24 h, the cultures' supernatants were subjected to HPLC, to determine residual sugar and metabolites produced. Table 3 shows that *L. fermentum* 92294 very efficiently fermented the sugars applied: under all conditions tested, only small amounts of glucose or fructose were detected in the supernatants, indicating that citrate or pyruvate did not (glucose plus citrate) or only marginally (fructose plus citrate, fructose plus pyruvate) interfere with fermentation. In the presence of glucose, pyruvate appeared to even enhance fermentation. *W. confusa* behaved quite differently: as seen before (Table 2), significant amounts of sugar remained at the end of fermentation (Table 3). Citrate appeared to enhance fermentation, especially in the presence of glucose, whereas pyruvate delayed fermentation of both sugars quite significantly. Finally, *S. cerevisiae* 56101 fermented both sugars quite efficiently only in the absence of pyruvate or citrate. Pyruvate delayed fermentation, whereas citrate inhibited growth of the yeast at all. The latter effect was confirmed by the trial in which cell concentrations and metabolites were analyzed (Figure 3): in the presence of citrate, *S. cerevisiae* 56101 did not grow (cell numbers remained or fell slightly below the number of ca. 10^6^ cfu/ml at inoculation) and did not produce any metabolite. Chen et al. (2010) showed that supplementation of growth medium of *S. cerevisiae* with 1 g sodium citrate per liter resulted in suppressed phosphofructokinase activity during fermentation. Higher concentrations, however, may result in acidification of the cytosol of the yeast and may be responsible for growth inhibition. Pyruvate, on the other hand, is effectively taken up by *S. cerevisiae* into the cytoplasm (Akita et al., 2000), where it may enter the carbohydrate and energy-yielding metabolism (Flores et al., 2000). Intracellular accumulation of pyruvate has been shown to result in simultaneous formation of ethanol and acetate (Van Urk et al., 1990; van Dijken et al., 1993). Glycerol production is one way of NAD^+^ regeneration and occurs mainly when NADH is produced in reactions different from glycolysis (Flores et al., 2000). The increased variability of metabolites, when pyruvate was added, corresponds with previous observations (Zelle et al., 2008).

**Table 3:** **Concentration of residual sugar at the end of fermentation (37°C, 24 h) by tested microorganisms**.

**Strains**	**Residual sugar (mM ± standard deviation) after fermentation in MCB medium containing:**					
	**Glucose**	**Glucose + Citrate**	**Glucose + Pyruvate**	**Fructose**	**Fructose + Citrate**	**Fructose + Pyruvate**
*L. fermentum* 92294	5.8±3.5	6.3±2.5	0.0±0.0	0.0±0.0	5.6±3.3	5.3±2.5
*W. confusa* NRRL-B- 14171	33.7±2.6	0.0±0.0	77.5±2.6	35.1±1.9	22.8±1.7	70.1±3.5
*S. cerevisiae* 56101	12.1±2.6	82.3±1.5	42.3±1.9	24.3±2.5	83.0±1.7	45.9±2.5

*Fermentation medium contained 83.3 mM hexose*.

Analyses of metabolites produced by *L. fermentum* 92294 and *W. confusa* NRRL-B-14171 (Figure 3) showed that addition of citrate and pyruvate significantly affected amounts and profiles of metabolites. *L. fermentum* 92294 produced mainly lactate and ethanol when grown on glucose, but produced lactate, erythritol, and acetate in about equimolar concentrations of ca. 50–70 mM at the expense of ethanol when grown on glucose plus citrate. Ethanol concentration was reduced from ca. 70 mM to < 10 mM (Figure 3). When pyruvate was added in addition to glucose, the dominating metabolite produced was lactate. Its concentration was almost twice as high as in the presence of glucose alone. Acetate increased from ca. 5 to 25 mM, whereas ethanol decreased from ca. 70 to 20 mM. Acetaldehyde was clearly detected, however in small amount. When fructose was applied as carbon source for *L. fermentum* 92294, a considerable part of fructose was immediately reduced to mannitol. Addition of citrate resulted in reduced amounts of mannitol and increased amounts of lactate, erythritol, acetate, and ethanol, with erythritol becoming the dominating metabolite (Figure 3). When pyruvate was added in addition to fructose, the most important change in metabolites was the increase in lactate. No erythritol and only little amounts of ethanol were produced under these conditions. Again, a small but clearly detectable amount of acetaldehyde was seen.

When *W. confusa* NRRL-B-14171 was applied for fermentation, no difference in metabolites profiles was seen upon growth on glucose and fructose, respectively. Lactate and ethanol were produced in almost equimolar amounts of ca. 45 μmoles/ml and only tiny amounts of acetate (<5 μmoles/ml) were detected (Figure 3). Addition of citrate resulted in increased production of lactate (90–100 μmoles/ml) as well as acetate (ca. 75 and 40 μmoles/ml) from glucose and fructose, whereas decreased but still significant production of ethanol was observed from both sugars (20 and 25 μmoles/ml from glucose and fructose, respectively). When pyruvate was added instead of citrate, ethanol production was further decreased to < 5 mM concentrations and small concentrations between 5 and 10 mM of acetaldehyde were detected. Lactate and acetate were the predominant metabolites.

These results confirm the data of Zaunmüller et al. (2006), who showed that hetero-fermentative LAB can use fructose, citrate, and pyruvate as external electron acceptors for regeneration of NAD(P)H, thus circumventing NAD(P)H regeneration by ethanol production, which appears to be the limiting step in hexose fermentation. Citrate is found in high concentrations in citrus fruits (up to 260 mM; Penniston et al., 2008) and in rather low concentrations in milk (ca. 8 mM; Linzell et al., 1976). It has many applications in food industry, where it is e.g., used as an acidulant in many different foods (Stratford, 1999) or as emulsifier in production of processed cheese (Zehren and Nusbaum, 2000). Pyruvate is present in high concentrations in red apples (ca. 450 mg per average sized red apple; https://pubchem.ncbi.nlm.nih.gov/compound/Pyruvic_acid#section=Drug-and-Medication-Information) and can be used as antioxidant, free radical scavenger, anti-inflammatory agent, and precursor for biosynthesis of amino acids like tryptophan and alanine (Li et al., 2001; Venkataraman et al., 2002; Aarnikunnas et al., 2003; Song et al., 2004).

### Modulation of fructose fermentation in fecal slurries by addition of defined microorganisms

To study *in vitro* the influence of fecal microbiota by specific microorganisms and dietary electron acceptors, slurries were prepared from fecal samples. Fructose was added as carbon source for fermentation. Feces samples were obtained from four healthy, overweight, male volunteers. The fresh samples were investigated for some major microbial groups: total anaerobes, *Enterobacteriaceae*, lactobacilli, and clostridia. The total number of anaerobes considerably varied between the samples by more than two orders of magnitude (Table 4). Lactobacilli represented between 0.01 and 10% of the numbers of total anaerobes. Numbers of clostridia reached only 10% of the numbers of lactobacilli. *Enterobacteriaceae* were present in lowest numbers reaching just between 0.01 and 1% of those of lactobacilli.

**Table 4:** **Microbiological analysis of fresh feces samples collected from four overweight volunteers**.

**Volunteers**	**Log cfu/g** ± **standard deviation**			
	**Anaerobes**	***Enterobacteriaceae***	**Lactobacilli**	**Clostridia**
1	13.0 ± 0.4	6.3 ± 0.5	9.4 ± 0.3	8.4 ± 0.4
2	12.5 ± 0.6	6.6 ± 0.4	8.6 ± 0.5	7.7 ± 0.6
3	10.5 ± 0.7	5.3 ± 0.4	9.4 ± 0.3	7.4 ± 0.5
4	11.7 ± 0.6	6.5 ± 0.4	10.3 ± 0.5	9.6 ± 0.4

*±, standard deviation*.

The rational for investigating with the slurries prepared from fecal samples which metabolites were produced from fructose, was based on the role fructose is supposed to play in the development of NAFLD (Machado and Cortez-Pinto, 2012). The increase of fructose consumption in recent years is considered to be a risk for NAFLD (Ouyang et al., 2008) and its metabolism apparently enhances triglyceride synthesis resulting in *de novo* lipogenesis (Lim et al., 2010). The microorganisms chosen to be added to the slurries were all from the phylum *Firmicutes*, which had been shown to be increased in the gut microbiota of obese humans (Turnbaugh et al., 2006): *A. caccae DSM 14662* was used as a control not producing ethanol, and *L. acidophilus* NRRL B-4495, *L. fermentum* 92294, and *W. confusa* NRRL-B-14171 representing homo-fermentative, hetero-fermentative *mdh*-expressing, and hetero-fermentative non-*mdh*-expressing lactic acid bacteria, respectively. In addition, citrate and pyruvate were added as dietary electron acceptors and neomycin to control for microbiota-dependent metabolite production.

Table 5 shows that of the metabolites analyzed (lactate, acetate, propionate, butyrate, mannitol, ethanol) in fecal slurries mannitol was detected in highest concentrations of ca. 11 mg/ ml, followed by lactate (ca. 5 mg/ml) and acetate (ca. 1.6 mg/ml). Ethanol was found in all four slurries with a mean concentration of ca. 1 mg/ml. Propionate was consistently found in low concentrations of ca. 0.7 mg/ml, whereas butyrate was found in only one slurry at a concentration of 0.6 mg/ml. Since mannitol dehydrogenase activity is mainly found in lactic acid bacteria, yeasts, and fungi (Saha and Racine, 2011), the high prevalence of mannitol and lactate appears to indicate a high abundance of lactic acid bacteria expressing mannitol dehydrogenase activity. Addition of neomycin significantly reduced mannitol, ethanol, propionate, butyrate, and acetate production. Lactate production, however, was not affected at all. A possible explanation could be that due to inhibition of mannitol dehydrogenase-expressing lactic acid bacteria, neomycin-insensitive lactic acid-producing bacteria like e.g., bifidobacteria not expressing mannitol dehydrogenase could take over fermentation. Although neomycin is active against Gram-negative and Gram-positive bacteria, insensitivity against this antibiotic is found in several lactic acid bacteria and in bifidobacteria in particular (D'Aimmo et al., 2007; Ashraf and Shah, 2011; Abriouel et al., 2015).

**Table 5:** **Metabolites produced from fructose by differently treated faecal slurries**.

**Treatment**	**Metabolite concentration [mg/ml]**^a^					
	**Lactate**	**Acetate**	**Propionate**	**Butyrate**	**Mannitol**	**Ethanol**
Faecal slurry (Fs)	6.5 ± 0.8/3.5 ± 0.7	2.3 ± 0.4/0.8 ± 0.4	0.9 ± 0.4/0.4 ± 0.2	0.6 ± 0.1/<0.1	13.6 ± 0.4/8.1 ± 0.3	1.3 ± 0.3/0.8 ± 0.3
Fs + neomycin	5.4 ± 0.7/4.4 ± 0.6	0.6 ± 0.1/0.3 ± 0.1	<0.1	<0.1	1.8 ± 0.5/1.6 ± 0.4	<0.1
Fs + *A. caccae*	3.1 ± 0.6/2.3 ± 0.9	1.3 ± 0.3/0.7 ± 0.3	0.6 ± 0.2/<0.1	2.9 ± 0.2/1.4 ± 0.3	1.8 ± 0.4/1.6 ± 0.5	<0.1
Fs + *L. acidophilus*	6.5 ± 0.9/5.5 ± 0.7	2.3 ± 0.4/1.7 ± 0.3	0.5 ± 0.2/0.2 ± 0.1	<0.1	1.5 ± 0.3/1.4 ± 0.3	<0.1
Fs + *L. fermentum*	3.6 ± 0.7/2.8 ± 0.7	1.7 ± 0.3/1.3 ± 0.4	0.2 ± 0.1/<0.1	0.6 ± 0.1/<0.1	11.5 ± 1.0/8.9 ± 0.6	0.3 ± 0.1/<0.1
Fs + *W. confusa*	5.9 ± 0.4/5.3 ± 0.3	1.8 ± 0.2/0.7 ± 0.3	0.2 ± 0.0/<0.1	<0.1	0.2 ± 0.1/<0.1	5.6 ± 0.4/3.1 ± 0.4
Fs + citrate	12.1 ± 0.9/10.6 ± 0.9	7.8 ± 0.5/6.0 ± 0.6	0.9 ± 0.4/<0.1	<0.1	0.1 ± 0.0/<0.1	<0.1
Fs + pyruvate	19.3 ± 2.1/12.4 ± 0.8	3.1 ± 0.2/1.8 ± 0.5	1.9 ± 0.5/1.8 ± 0.5	<0.1.	0.2 ± 0.1/<0.1	<0.1

a *Highest/lowest mean value ± standard deviation of slurries from four volunteers are shown*.

Addition of *A. caccae DSM 14662* resulted in suppressed production of propionate, mannitol, and ethanol, similar as seen with addition of neomycin. Lactate was even higher and acetate somewhat less suppressed as compared to neomycin. Butyrate production, however, was considerably higher than with untreated slurries. The latter is consistent with *A. caccae*'s metabolism, which is known to produce mainly butyrate from hexoses (Schwiertz et al., 2002; Duncan et al., 2004). Apparently, addition of comparably high numbers of *A. caccae DSM 14662* to the slurries resulted in suppression of many bacteria involved in production of metabolites in untreated slurries.

When *L. acidophilus* NRRL B-4495 was added to the slurries, the most pronounced effect seen in comparison to untreated slurries was for mannitol: production was reduced to ca. 1.5 mg/ml. Ethanol and butyrate were not detected, propionate was somewhat reduced, and lactate and acetate levels remained unaffected. This indicates that addition of high numbers of this homo-lactic bacterium suppressed to a large extent mannitol-producing hetero-lactic bacteria in the slurries.

When *L. fermentum* 92294, a hetero-lactic mannitol-producing lactic acid bacterium was added, the mannitol level remained basically the same as in untreated slurries. Surprisingly, lactate production was reduced to ca. half of that in untreated slurries. This is in contrast to fermentation in MCB-medium, where mannitol and lactate were produced in ca. equal amounts (Figure 3A). However, it agrees with earlier observations that under different growth conditions mannitol and other metabolites are produced in varying proportions (Wisselink et al., 2002). Furthermore, one has to take into account that in a slurry-system co-fermentation takes place, thus, metabolites produced by some organisms may be metabolized by others.

**Figure 4:**
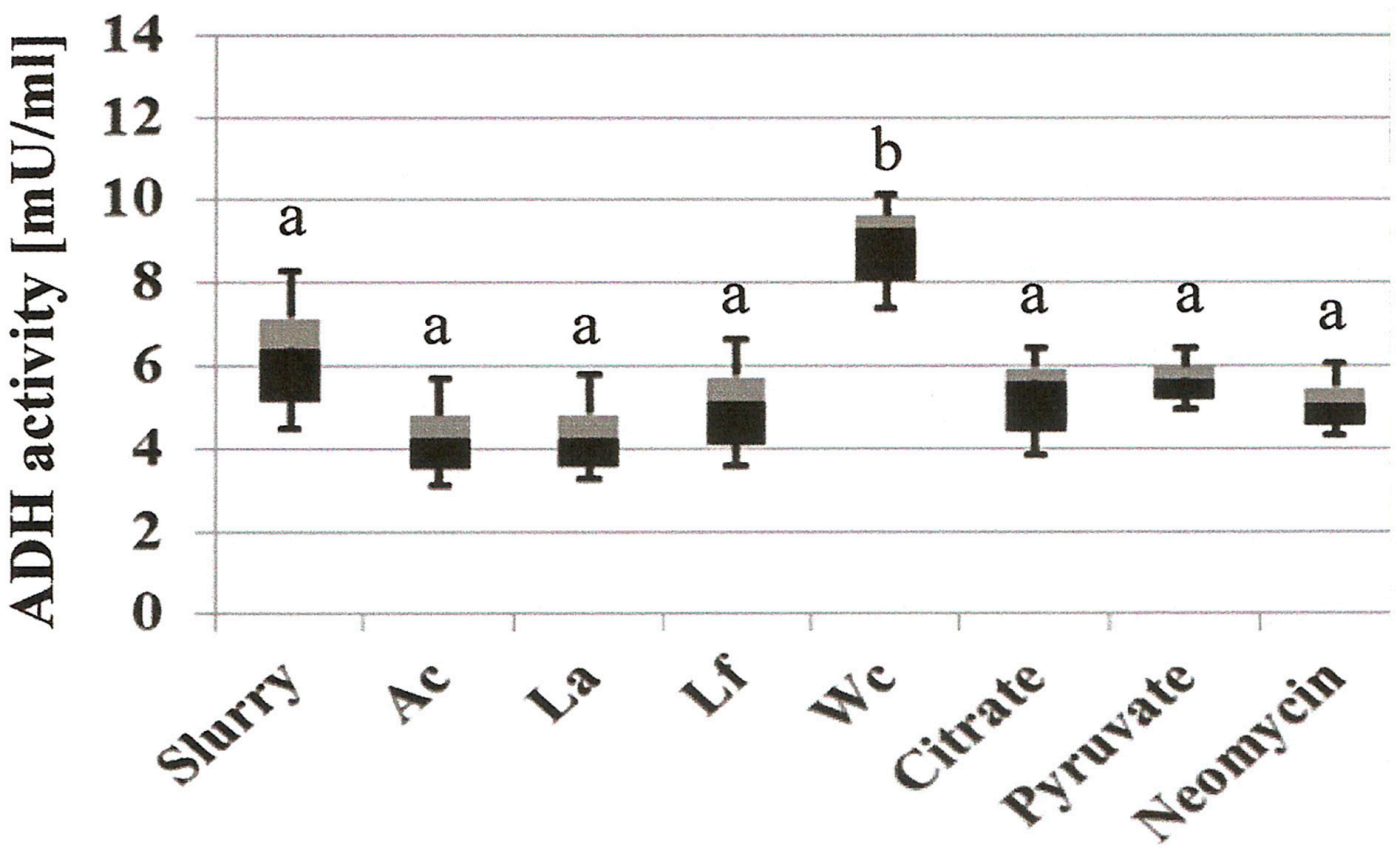
**Microbial alcohol dehydrogenase activity (ADH, mU/mL) of differently treated fecal slurries after 24 h incubation at 37°C in MCB containing fructose**. A box-and whisker plot is shown, with the median indicated by the border of the black and gray box and the whiskers indicating maximal and minimal values. Slurry, without added bacterial strain; Ac, *A. caccae* DSM 14662; La, *L. acidophilus* NRRL-B-4495; Lf, *L. fermentum* 92294; Wc, *W. confusa* NRRL-B-14171. Different letters (a and b) indicate significant differences (*P* < 0.05) in *post-hoc* Tuckey test.

Addition of *W. confusa* NRRL-B-14171 showed one remarkable alteration with respect to untreated slurries, i.e., alcohol production was significantly (*P* < 0.05) increased by a factor of 3–4. This effect was confirmed by measuring alcohol dehydrogenase (ADH) activities in the differently treated slurries (Figure 4). Addition of *W. confusa* NRRL-B-14171 caused an increase in ADH activity that was significantly (*P* < 0.05) different to all other treatments. Finally, addition of citrate and pyruvate, respectively, suppressed ethanol and mannitol production while stimulating lactate and acetate production. Pyruvate as a direct electron-acceptor for NADH significantly stimulated lactate production, whereas citrate as a precursor molecule for acetyl-CoA showed considerable increase in acetate production besides increased lactate production. These data appear to indicate that both dietary electron-acceptors favorably affect mostly non-mannitol-producing hetero-lactic bacteria residing in the microbiotas of the slurries.

## Conclusions

Our data show that significant amounts of ethanol were produced *in vitro* from glucose and fructose by *L. fermentum* 92294, *W. confusa* NRRL-B-14171, and *S. cerevisiae* 56101. Addition of pyruvate resulted in suppressed production of ethanol from glucose and fructose in all three organisms, whereas citrate was only effective in *L. fermentum* 92294 and *W. confusa* NRRL-B-14171. In *S. cerevisiae*, addition of citrate resulted in growth inhibition. In fructose-fermenting fecal slurries prepared from feces samples of four healthy obese volunteers, addition of *A. caccae* DSM 14662, *L. acidophilus* NRRL B-4495, *L. fermentum* 92294, citrate, and pyruvate, respectively, resulted in suppressed ethanol production. Addition of *W. confusa* NRRL-B-14171, on the other hand, resulted in increased ethanol production. Our results show that under *in vitro* conditions glucose and fructose were the sugars yielding highest amounts of ethanol. The disaccharides and the polysaccharide tested did not yield comparable amounts of ethanol, although they consisted almost entirely of fructose (inulin), of fructose and galactose (lactulose), or of glucose and galactose (lactose). In correlation with the results on dietary electron-acceptors, these data show that by the choice of diet intestinal ethanol production may be suppressed. This is consistent with the concept of prebiotics, which are sugars or polysaccharides not metabolized by the host but by a fraction of the gut microbiota, whereby positive health effects on the host are exerted. We think that our experiments with fecal slurries had two interesting outcomes. (i) There was considerable ethanol produced from fructose by all four slurries prepared from feces samples of healthy overweight adults. This may indicate that indeed a lifestyle resulting in obesity may pave the way for NAFLD through dysbalanced microbiota producing ethanol. We have to bear in mind that we analyzed just four different feces samples and that we did not include control samples of normal weight subjects. However, we may take these results as a starting point for further experiments on a broader basis, with more volunteers and appropriate controls. (ii) *W. confusa* NRRL-B-14171 was the only one of the microorganisms tested that caused increased ethanol production. We do not think that indeed *W. confusa* is the one “bad bug” responsible for induction of NAFLD: for such a conclusion our data are based on too few microorganisms. However, we think that microorganisms with similar metabolism, like e.g., non-mannitol-producing hetero-lactic fermentation metabolism, may be involved in promoting NAFLD through continuous production of ethanol in the intestine. *W. confusa* belongs to the *Firmicutes*, which have been shown to be associated with NAFLD. We tested our hypothesis by feeding *W. confusa* NRRL-B-14171 to Wistar rats in addition to a high fat, high fructose diet. The data are described in an accompanying manuscript submitted to the same Research Topic of Frontiers in Microbiology.

## Author contributions

FE: conception, acquisition, analysis, and interpretation of data for the work. WB: conception, analysis, and interpretation of data for the work. DM: analysis, and interpretation of data for the work. Md: analysis, and interpretation of data for the work. HW: analysis, and interpretation of data for the work. JS: conception, design, interpretation of data for the work. KH: conception, design, interpretation of data for the work.

### Conflict of interest statement

The authors declare that the research was conducted in the absence of any commercial or financial relationships that could be construed as a potential conflict of interest.
